# Data-Driven Insights in Healthcare

**DOI:** 10.3390/healthcare13212658

**Published:** 2025-10-22

**Authors:** Victor R. Prybutok, Gayle L. Prybutok

**Affiliations:** 1 Department of Information Technology and Decision Sciences, G. Brint Ryan College of Business, University of North Texas, Denton, TX 76203, USA; 2 Department of Rehabilitation and Health Services, College of Health and Public Service, University of North Texas, Denton, TX 76203, USA

## 1. Introduction

We are pleased to present this Special Issue, which is a curated collection of research that showcases the transformative power of data-driven approaches in healthcare. The healthcare sector generates vast amounts of observational data daily, yet systematic exploration of these datasets to uncover meaningful patterns remains underutilized. The rapid advancement of digital health technologies, including electronic health records, medical imaging systems, wearable devices, and genomic sequencing platforms, has led to an exponential growth in healthcare data availability [1]. In addition, data from routine clinical practices offer unique opportunities to complement evidence from randomized controlled trials, particularly for understanding treatment effectiveness in diverse patient populations and real-world clinical settings [2,3]. However, ensuring the quality and appropriate use of these observational datasets remains a persistent challenge that requires systematic attention [4,5]. The collection in this Special Issue demonstrates that while theory-driven research remains essential, data exploration can generate hypotheses, reveal unexpected associations, and provide evidence that directly informs practice and future research directions.

The ten contributions assembled in this Special Issue span diverse healthcare contexts and analytical methodologies, unified by a commitment to extracting actionable knowledge from empirical observations. These publications employ innovative techniques, including artificial intelligence, machine learning, natural language processing, advanced statistical modeling, operations research, and exploratory data analytics, to address critical challenges in healthcare delivery, quality improvement, and patient outcomes [6]. The integration of these data-driven methodologies supports the need for future research and application regarding how healthcare systems can leverage observational evidence to inform clinical decision-making and policy development [7].

## 2. Artificial Intelligence and Machine Learning for Clinical Prediction

The Special Issue opens with three contributions that demonstrate the power of artificial intelligence and advanced analytical methods for clinical prediction and decision support. Halwani and Halwani (contribution 1) present “Prediction of COVID-19 Hospitalization and Mortality Using Artificial Intelligence,” employing decision trees, support vector machines, and random forest algorithms to predict hospital mortality among COVID-19 patients. Their analysis of data from King Abdulaziz University Hospital in Saudi Arabia achieved predictive accuracy rates of 76–82%, with hospital stay duration, D-Dimers, alkaline phosphatase, bilirubin, lactate dehydrogenase, C-reactive protein, and ferritin identified as significant mortality predictors. This work illustrates how AI tools can enhance early identification of high-risk patients and support clinical decision-making during pandemic situations.

Alasmari (contribution 2) provides a comprehensive scoping review titled “A Scoping Review of Arabic Natural Language Processing for Mental Health,” examining NLP techniques applied to mental health detection in Arabic-speaking populations. Following the PRISMA-ScR framework, this review identifies the effectiveness of various approaches, with transformer-based models such as AraBERT and MARBERT achieving superior performance with accuracy rates up to 99.3% and 98.3%, respectively. The review highlights how NLP can analyze social media data to detect depression and suicidality, demonstrating the potential of these techniques for population-level mental health surveillance in linguistically diverse contexts.

Chang, Ryu, Choi, Kwon, and Kim (contribution 3) present “A Comparative Study of Hospitalization Mortality Rates between General and Emergency Hospitalized Patients Using Survival Analysis,” employing Kaplan–Meier survival estimation and Cox proportional hazards models to analyze four years of data from the Korean National Health Insurance Services. Their analysis reveals distinct determinants of mortality risk between general inpatients and emergency admissions, with geographic factors and institutional characteristics such as physician and nurse staffing ratios, bed capacity, and emergency bed availability showing differential effects. This work demonstrates how survival analysis techniques can accommodate censored medical data characteristics often overlooked by conventional regression approaches.

## 3. Data-Driven Quality Improvement and Healthcare Standards

Two articles explore how data-driven methodologies can enhance healthcare quality standards and establish evidence-based benchmarks. Richardson, Penumaka, Smoot, Panaganti, Chinta, Guduri, Tiyyagura, Martin, Korvink, and Gunn (contribution 4) present “A Data-Driven Approach to Defining Risk-Adjusted Coding Specificity Metrics for a Large U.S. Dementia Patient Cohort,” analyzing 487,775 hospitalization records to develop risk-adjusted metrics for assessing medical coding specificity. Using logistic regression models incorporating patient and facility characteristics, combined with Poisson binomial modeling, they created benchmarks enabling healthcare facilities to assess coding practices against industry standards. With an AUC of 0.727 for principal dementia diagnoses, their approach demonstrates how data-driven methods can identify facilities that over- or under-specify diagnoses, ultimately contributing to improved patient care quality and healthcare system reliability.

Velev, Velazquez-Sosa, Lebien, Janwa, and Roche-Lima (contribution 5) provide “Modeling Multivariate Distributions of Lipid Panel Biomarkers for Reference Interval Estimation and Comorbidity Analysis,” employing Gaussian Mixture Models to derive reference intervals directly from large-scale, real-world laboratory data from Puerto Rico. Their methodology enables separation of healthy and pathological subpopulations without relying on diagnostic codes, producing sex- and age-stratified reference intervals for total cholesterol, LDL, HDL, and triglycerides. By examining selective mortality patterns and constructing comorbidity implication networks, they explain counterintuitive age trends in lipid values and characterize interdependencies between conditions, demonstrating how population-specific reference intervals can be derived without recruiting healthy cohorts.

## 4. Healthcare Operations Research and Resource Optimization

Two contributions employ operations research methodologies to optimize healthcare resource allocation and workforce management. Mystakidis, Koukaras, Koukaras, Kaparis, Stavrinides, and Tjortjis (contribution 6) present “Optimizing Nurse Rostering: A Case Study Using Integer Programming to Enhance Operational Efficiency and Care Quality,” developing a comprehensive integer programming model for nurse scheduling in oncology departments. Their model integrates constraints including legal work hours, staff qualifications, and personal preferences to generate equitable and efficient schedules. Implementation in a clinical setting revealed significant improvements in scheduling efficiency, staff satisfaction, workload distribution, and compliance with work-hour regulations, demonstrating how operations research techniques can enhance both operational excellence and staff well-being in acute care settings.

Clapper, ten Hove, Bekker, and Moeke (contribution 7) provide “Team Size and Composition in Home Healthcare: Quantitative Insights and Six Model-Based Principles,” developing six model-based principles to guide managerial decisions regarding home healthcare team structure. Through extensive data analysis and mathematical modeling based on real-life scenarios, they demonstrate that efficiency improves with team size but with diminishing returns, while team manageability becomes increasingly complex as size grows. Their work provides estimates for travel time based on team size and territory, establishes upper bounds for full-time contract fractions to avoid split shifts, and concludes that ideally sized teams should serve at least several hundred care hours weekly. This research exemplifies how quantitative modeling can inform practical workforce planning decisions.

## 5. Technology-Enabled Healthcare and Behavioral Insights

Two articles examine how technology shapes health-seeking behaviors and social interactions in healthcare contexts. Boyce, Harun, G. Prybutok, and V. Prybutok (contribution 8) present “The Role of Technology in Online Health Communities: A Study of Information-Seeking Behavior”, employing partial least squares structural equation modeling with multi-group and importance-performance map analysis to examine technology’s role in online health communities. Their cross-sectional survey identifies ease of site navigation and interaction with other members as the most beneficial technology-related factors influencing information-seeking processes. The findings provide actionable insights for developing and managing online health communities and for healthcare professionals seeking to disseminate relevant information to individuals with chronic illnesses such as COPD.

Chen, Hsu, and Rahman (contribution 9) provide “From Mandate to Choice: How Voluntary Mask Wearing Shapes Interpersonal Distance Among University Students After COVID-19,” examining the association between voluntary protective behaviors and social interactions in post-mandate Taiwan. Through an online interpersonal distance simulation with 100 university students, they employed four-way ANOVA to reveal that mask-wearing individuals maintain significantly greater interpersonal distances, suggesting heightened risk perception, while masked targets elicit smaller distances, possibly due to safety signaling. Gender differences emerged in both protective behavior adoption (72% of females versus 44% of males) and spatial preferences, offering insights into how voluntary behavioral adaptations continue shaping social norms after mandate removal.

## 6. Research Infrastructure and Data Governance

The Special Issue concludes with a perspective on research infrastructure challenges. Landi, D’Ambrosio, Faggion, Rocchi, Paganin, Lain, Ceci, and Giannuzzi on behalf of the EPIICAL Consortium (contribution 10) present “Sharing Data and Transferring Samples Within Pediatric Clinical Studies: How to Overcome Challenges and Make Them a Science Opportunity.” This perspective examines the EPIICAL project’s establishment of a dedicated Working Group to navigate ethical and regulatory complexities of international pediatric clinical studies involving HIV-infected children. The consortium developed well-structured informed consent and assent templates, data sharing agreements, and material transfer agreements to regulate sample transfers among partners and sites across European and non-European boundaries. This contribution highlights how structured governance frameworks and expert support can transform regulatory challenges into opportunities for advancing pediatric clinical research.

## 7. Conclusions

The contributions assembled in this Special Issue collectively demonstrate that data-driven discovery in healthcare extends far beyond descriptive analysis. These works show how systematic exploration of observational data using diverse methodologies, from artificial intelligence and machine learning to operations research and behavioral modeling, can generate actionable insights that improve patient care, enhance operational efficiency, establish evidence-based standards, and inform policy decisions.

As healthcare systems continue generating unprecedented volumes of data through digital transformation initiatives [1,6], the approaches showcased in this Special Issue provide both inspiration and practical guidance for researchers, practitioners, and policymakers seeking to extract maximum value from their data assets. The successful translation of data-driven insights into improved patient outcomes requires not only sophisticated analytical methods but also robust data governance frameworks, quality assurance mechanisms, and ethical oversight [2,4]. We hope this collection serves as a catalyst for continued innovation in data-driven healthcare research and practice.
